# Assessment of QRISK3 as a predictor of cardiovascular disease events in type 2 diabetes mellitus

**DOI:** 10.3389/fendo.2022.1077632

**Published:** 2022-11-28

**Authors:** Xiaodie Mu, Aihua Wu, Huiyue Hu, Hua Zhou, Min Yang

**Affiliations:** Department of Nephrology, The Third Affiliated Hospital of Soochow University, Changzhou, China

**Keywords:** cardiovascular disease, type 2 diabetes mellitus (DM), risk score, QRESEARCH risk estimator version 3 (QRISK3), framingham risk score (FRS)

## Abstract

**Background:**

The risk of cardiovascular disease (CVD) in diabetes mellitus (DM) patients is two- to three-fold higher than in the general population. We designed a 10-year cohort trial in T2DM patients to explore the performance of QRESEARCH risk estimator version 3 (QRISK3) as a CVD risk assessment tool and compared to Framingham Risk Score (FRS).

**Method:**

This is a single-center analysis of prospective data collected from 566 newly-diagnosed patients with type 2 DM (T2DM). The risk scores were compared to CVD development in patients with and without CVD. The risk variables of CVD were identified using univariate analysis and multivariate cox regression analysis. The number of patients classified as low risk (<10%), intermediate risk (10%-20%), and high risk (>20%) for two tools were identified and compared, as well as their sensitivity, specificity, positive and negative predictive values, and consistency (C) statistics analysis.

**Results:**

Among the 566 individuals identified in our cohort, there were 138 (24.4%) CVD episodes. QRISK3 classified most CVD patients as high risk, with 91 (65.9%) patients. QRISK3 had a high sensitivity of 91.3% on a 10% cut-off dichotomy, but a higher specificity of 90.7% on a 20% cut-off dichotomy. With a 10% cut-off dichotomy, FRS had a higher specificity of 89.1%, but a higher sensitivity of 80.1% on a 20% cut-off dichotomy. Regardless of the cut-off dichotomy approach, the C-statistics of QRISK3 were higher than those of FRS.

**Conclusion:**

QRISK3 comprehensively and accurately predicted the risk of CVD events in T2DM patients, superior to FRS. In the future, we need to conduct a large-scale T2DM cohort study to verify further the ability of QRISK3 to predict CVD events.

## Introduction

Diabetes mellitus (DM) has become more widespread owing to its high prevalence and related disability, estimated to affect 693 million individuals by 2045 (1, 2). The most challenging aspect of treatment is controlling diabetes-related complications. Macrovascular complications (cardiovascular and cerebrovascular diseases) and microvascular illness (diabetic nephropathy) lead to a significant increase in care expense, hospitalization frequency, mortality and a decline in quality of life (1, 3, 4). The risk of cardiovascular disease (CVD) was reported to be two- to three-fold higher in people with DM (5, 6). The development of CVD in DM patients is often a complication with high mortality, which mainly manifests in the coronary system, aorta and cerebral artery (7). Uncoordinated vasoconstriction and dilation, platelet aggregation, and lipid deposition in the vessel wall resulting in hyperglycemia, hyperlipidemia, hyperviscosity, and hypertension, resulting in an exponential increase in the incidence of DM-related CVD (8). Blood glucose control and clinical risk variables by themselves are unable to anticipate the onset of vascular complications. The concern is to find alternative indicators to identify CVD patients at high risk of DM disease.

If the subject has not yet suffered a CVD event, the QRESEARCH risk estimator version 3 (QRISK3) algorithm can be used to estimate a person’s probability of suffering a fatal or non-fatal heart attack or stroke within the next 10 years (9, 10). Based on QRISK2, this algorithm was jointly created in 2017 by doctors and academics working for the UK National Health Service. Developers and researchers thoroughly verified the QRISK algorithm utilizing UK primary care databases such as QResearch and other large cohorts clinical studies (9, 11). In addition to the clinical factors already included in the QRISK2 risk prediction model, the QRISK3 risk prediction model includes additional clinical factors (severe mental illness (schizophrenia, bipolar disorder, moderate/severe depression), atypical antipsychotic use, corticosteroid use, systolic blood pressure variability measurements (standard deviation of repeated measurements), migraine, systemic lupus erythematosus (SLE), and erectile dysfunction) to assist physicians in identifying patients most at high risk for CVD events, early intervention, and treatment (9). There have been several previous studies on the effectiveness of QRISK3 in predicting CVD events in SLE and inflammatory bowel disease (IBD), but few studies on the performance of cardiovascular events in T2DM (12, 13).

A popular and well-known calculator, the Framingham risk score (FRS), is recommended in the American College of Cardiology/American Heart Association (ACC/AHA) practice guideline on the prevention of CVD disorders in clinical practice (14, 15). In addition to the CVD events listed in the original FRS model, the most recent version of the FRS, created in 2008, also includes transient ischemic attack (TIA) and cerebrovascular accident (CVA) (14). The FRS model incorporated multiple risk factors, such as age, sex, hypertension treatment, diabetes status, smoking status, high-density lipoprotein (HDL), total cholesterol, and systolic blood pressure (SBP), to estimate the 10-year risk of CVD (14). However, clinical factors related to CVD such as chronic kidney disease and family history were not included in the FRS compared to QRISK3.

To our knowledge, QRISK3 has not been tested in patients with T2DM in China. We tried to assess the application of QRISK3 for assessing CVD risk in individuals with type 2 diabetes mellitus (T2DM) in this present study. Since the FRS is a widely used CVD risk calculator, we compared the prediction performance of QRISK3 and the FRS to identify the presence of subclinical atherosclerosis in individuals with T2DM (16). Therefore, we designed a cohort trial in patients with newly-diagnosed T2DM for about 10 years, aiming to explore the effectiveness of QRISK3 and FRS as cardiovascular risk assessment tools, to best predict the development of CVD related to T2DM.

## Methods

### Study population

This was a retrospective analysis on prospectively collected T2DM patients data from the third affiliated hospital of soochow university. We selected 1003 newly-diagnosed T2DM patients in our hospital from December 2010 to September 2014 for long-term follow-up.

Inclusion criteria (1): meet the American Diabetes Association (ADA) classification criteria for T2DM and were newly-diagnosed patients (17) (2); age 25-84 years old (met the applicable range of QRISK3 algorithm).

Exclusion criteria (1): a history of CVD before enrolment in the cohort study (2); participation in clinical trials during the study period (3); incomplete clinical records.

The Framingham study in 2008, this study defined CVD events as (1) coronary heart disease (CHD) including coronary death, myocardial infarction (MI), coronary insufficiency and angina (2); atherosclerotic CVA including ischaemic stroke, haemorrhagic stroke and TIA (3); peripheral artery disease (PAD) secondary to atherosclerosis (intermittent claudication) (4); heart failure secondary to atherosclerosis (14).

We need to collect relevant data, including baseline data and information on cardiovascular risk factors (hypertension, smoking status, family history, psychiatric history) for calculating risk scores. The first CVD events identified throughout the follow-up period classified patients as ‘CVD patients’, while patients without CVD events were classified as ‘Non-CVD patients’.

### CVD risk algorithms

Although the CVD risk algorithm tools have relatively similar components, their efficacy may vary since they utilize various risk derivation algorithms and the same components have varying weights. For example, Age accounts for a high weight in the FRS model, but lacks indicators related to cardiovascular risk factors such as family history and chronic kidney disease history. In this study, QRISK3 and FRS established three categories of risk: low risk (<10%), intermediate risk (10-20%), and high risk (>20%).

FRS: includes age, sex, treatment for hypertension, DM status, smoking status, HDL, total cholesterol and systolic blood pressure (14).

QRISK3: includes age, sex, ethnicity, smoking status (non-smoker, ex-smoker, light smoker, moderate smoker, heavy smoker), DM status, family history of CVD (angina or heart attack in a first-degree relative younger than 60), chronic kidney disease, atrial fibrillation, blood pressure treatment, migraine, rheumatoid arthritis (RA), SLE, severe mental illness (schizophrenia, bipolar disorder, moderate/severe depression), atypical antipsychotic medication, steroid tablets use, diagnosis or treatment of erectile dysfunction, Cholesterol/HDL ratio, SBP and standard deviation of repeated blood pressure, height and weight.

### Data collection

The date of collection of baseline data and associated clinical data necessary to calculate the risk score was the ‘baseline date’. The ‘baseline date’ was defined as the date of the first visit to our hospital due to T2DM. Baseline clinical data was from the electronic medical record system, while clinical records (such as drug use history and family history) required to calculate the risk score and the CVD outcome are collected through follow-up.

### Statistical analysis

All statistical analysis was performed with SPSS 25.0. The median (interquartile range) [M (P25, P75)] was used to represent data with a non-normal distribution, whereas the mean ± standard deviation (SD) was used to express data with a quantitative normal distribution. The qualitative variable was selected as percentages (%). *P* < 0.05 was considered statistically significant.

The CVD risk score for each patient was calculated at the ‘baseline date’ using QRISK3 and FRS and assessed based on whether CVD occurred at the 10-year point. The low-risk (<10%), intermediate-risk (10%-20%) and high-risk (>20%) patient numbers of the two tools were identified and compared, and their sensitivity, specificity, positive predictive values (PPV), negative predictive values (NPV) and concordance (C) statistics were reported. Creating a proportional risk model (cox regression) to evaluate the correlation between QRISK3 and CVD. In addition, for each risk level, we estimated diagnostic hazard ratio (HR). C-statistics was used to assess the discriminative of each tool, where C-statistics was the area under the curve (AUC) of the receiver operating characteristics (ROC) curve with observed CVD as the outcome. C-statistics of 0.5 suggests no discrimination, 0.5-0.7 is considered acceptable, 0.7-0.9 excellent and greater than 0.9 outstanding (18). Kappa statistics were used to observe the similarities in total risk, low risk and high risk categories assigned by each tool. In addition, sensitivity analysis was carried out for the patients who lost to follow-up. Patients with loss to follow-up were included in the non-CVD and CVD groups, respectively, to compare whether there were differences in the conclusions.

## Results

**Figure 1:**
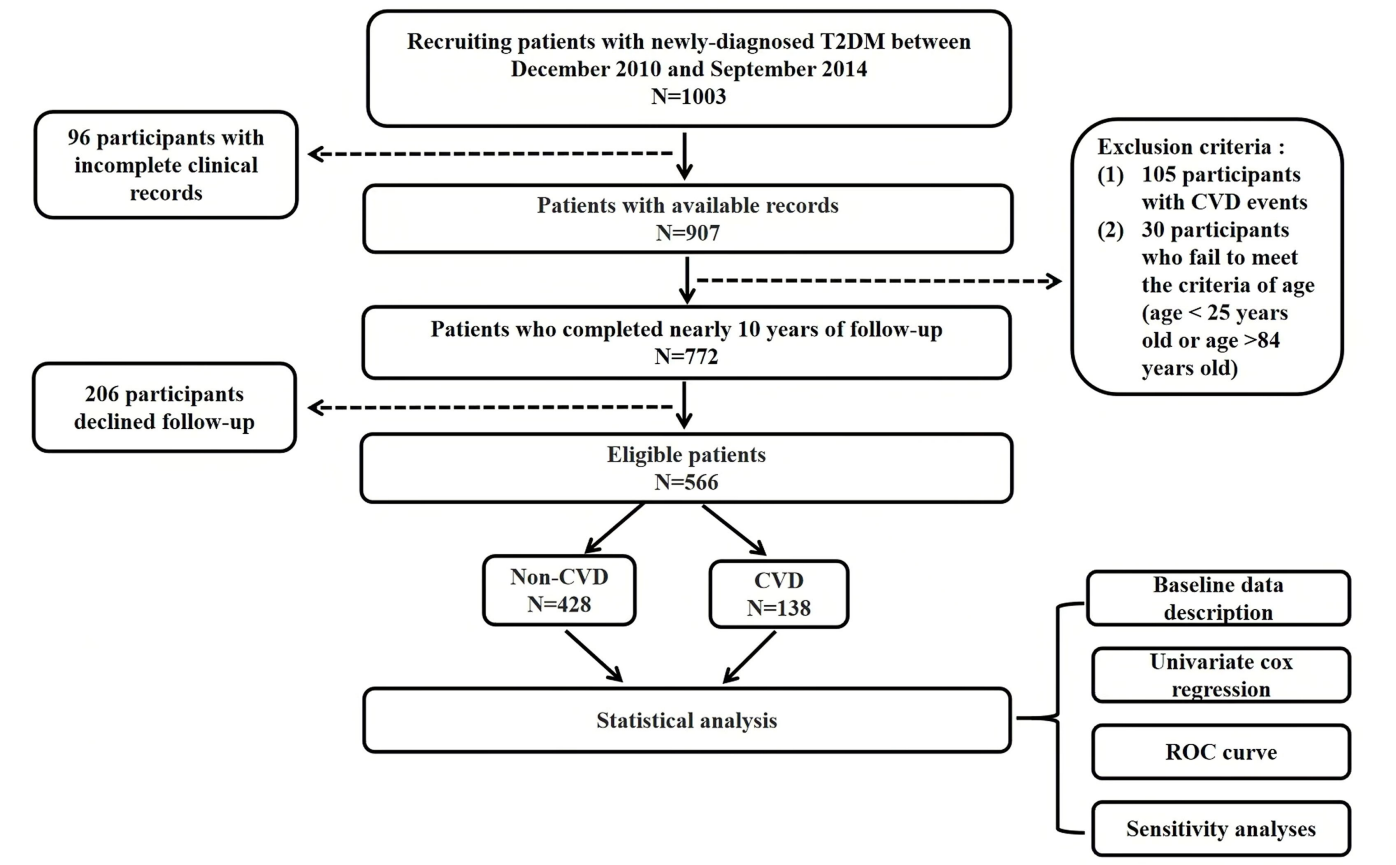
Flow chart of study. Legend: The flow chart shows the entire research process. T2DM, Type 2 diabetes mellitus; CVD, Cardiovascular disease; ROC, Receiver operating characteristics.

**Figure 2:**
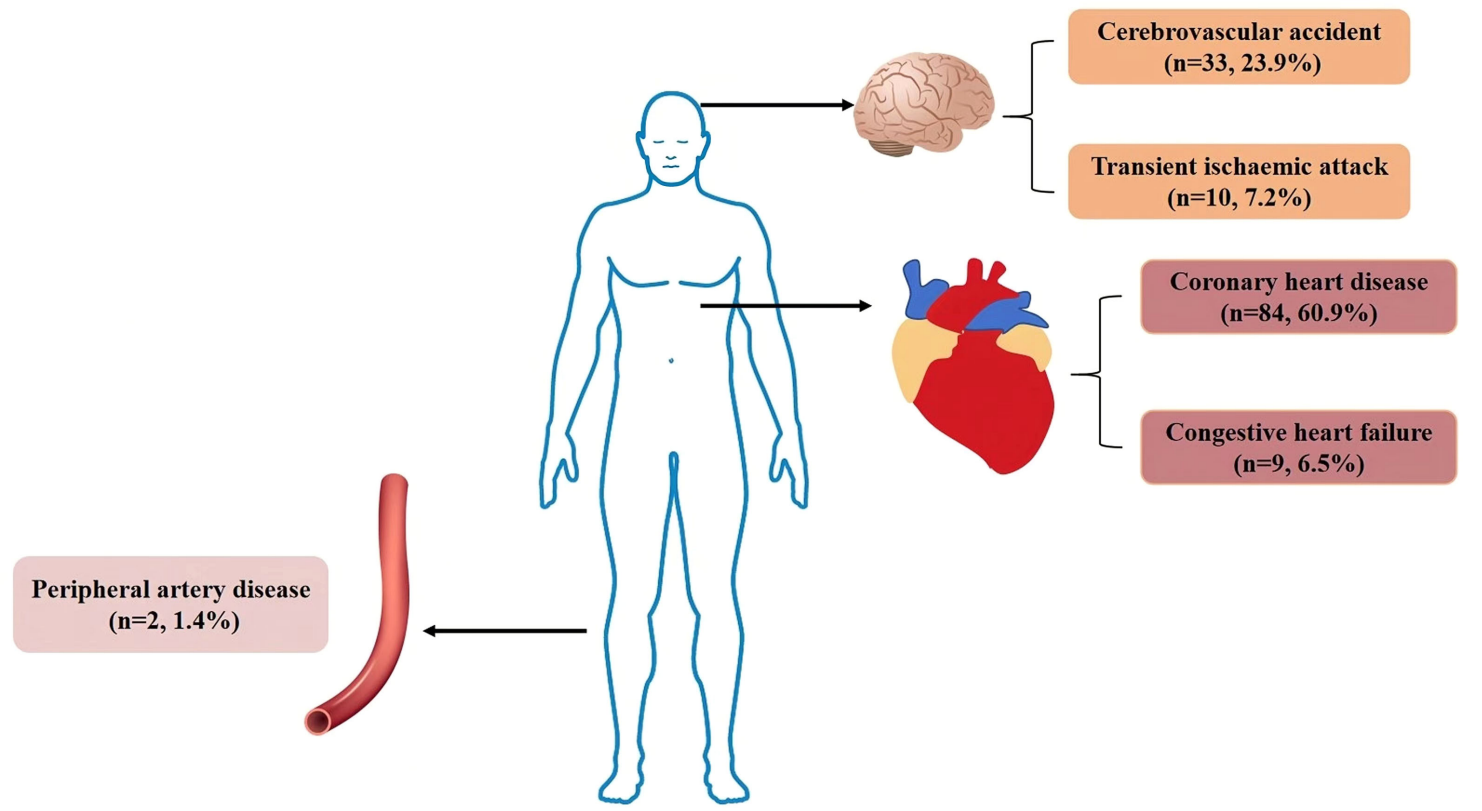
Classification of CVD events. Legend: In our cohort, there were 138 CVD events, 84 patients with coronary heart disease, 9 patients with congestive heart failure, 33 patients with cerebrovascular accident, 10 patients with transient ischaemic attack and 2 patients with peripheral arterial disease. CVD, Cardiovascular disease.

Out of the 1003 individuals in the cohort, 206 individuals were excluded due to loss of follow-up, 96 individuals were excluded due to insufficient clinical data, and 135 individuals were excluded due to ineligibility (105 individuals had CVD events prior to enrolment, and 30 individuals did not meet the age criteria). The final queue size was 566 persons (**Figure 1**). The average follow-up time of the T2DM clinical registration cohort was 8.68 ± 1.86years. There were 138 CVD events among the 566 individuals identified in our cohort. Of the 138 CVD events identified in our cohort, 84 (60.9%) developed coronary heart disease (see **Figure 2** for classification of CVD events).

The specific demographic and clinical characteristics of 566 T2DM patients are given in **Table 1**. Particular variables of discrepant include CVD patients being on average older in age (*p*<0.001), having higher blood pressure (*p*<0.001), higher fast C-peptide (FCP) (*p*=0.029), higher triglyceride-glucose index (TyG index) (*p*=0.018) and TC/HDL (*p*=0.023), and greater proportion with a history of smoking (*p*<0.001) and hypertension (*p*<0.001).

**Table 1:** Demographic and clinical characteristics of patients with Non-CVD and CVD.

Group	Non-CVD (n = 428)	CVD (n = 138)	Total (n = 566)
Age (years)	47.3 ± 11.7	59.5 ± 10.1	50.3 ± 12.5
Sex (male, %)	260.0 (60.7)	101.0 (73.2)	361.0 (63.8)
BMI(kg/m^2^)	25.2 (23.2, 27.1)	25.4 (23.4, 27.2)	25.3 (23.2, 27.1)
Smoking, n (%)	152.0 (34.3)	72.0 (52.2)	224.0 (39.6)
Hypertension, n (%)	144.0 (33.6)	80.0 (58.0)	250.0 (44.2)
SBP (mmHg)	135.4 ± 16.7	141.3 ± 19.1	135.0 (125.0, 147.0)
FBG (mmol/L)	9.5 (7.9, 11.7)	9.8 (7.9, 12.4)	9.5 (7.9, 11.9)
PBG (mmol/L)	14.5 (11.7, 17.1)	14.9 ± 4.1	14.6 (11.7, 17.1)
HbA1c (%)	10.8 (9.0, 12.3)	10.6 ± 2.6	10.7 (8.9, 12.4)
FCP (ng/ml)	1.6 (1.1, 2.3)	1.9 (1.3, 2.5)	1.7 (1.2, 2.4)
WBC (10^9g/L)	6.2 (5.4, 7.4)	6.7 (5.7, 8.1)	6.4 (5.5, 7.6)
Hb (g/L)	143.0 (134.0, 152.3)	141.2 (132.0, 149.3)	143 (133, 152)
NLR	1.6 (1.2, 2.0)	1.6 (1.3, 2.1)	1.6 (1.2, 2.0)
FIB (g/L)	2.4 (2.2, 2.9)	2.7 (2.2, 3.0)	2.5 (2.2, 2.9)
D-dimer (ug/L)	130.0 (100.0, 250.0)	160.0 (100.0, 282.5)	140.0 (100.0, 260.0)
ALT (U/L)	25.0 (18.0, 43.0)	27.0 (17.0, 43.0)	26.0 (18.0, 43.0)
AST (U/L)	16.0 (12.0, 23)	15.0 (12.0, 22.0)	16.0 (12.0, 23.0)
ALB (g/L)	37.9 (35.4, 40.2)	36.4 (34.5, 39.2)	37.4 (35.0, 39.9)
Bicarbonate (mmol/L)	23.7 (22.2, 25.6)	24.3 ± 2.7	23.9 (22.2, 25.7)
BUN (mmol/L)	4.7 (3.8, 5.7)	5.5 (4.2, 6.5)	4.8 (3.9, 5.9)
SC r (µmol/L)	69.2 ± 18.2	74.5 ± 16.6	70.5 ± 15.7
UA (µmol/L)	262.8 (211.2, 320.3)	289.5 ± 85.6	269.8 (216.8, 325.6)
CysC (mmol/L)	0.7 (0.6, 0.9)	0.7 (0.6, 0.9)	0.7 (0.6, 0.9)
eGFR (ml/min/1.73 min)	109.5 (91.1, 132.6)	96.1 (79.0, 112.3)	104.8 (88.5, 129.2)
K (mmol/L)	4.3 (4.1, 4.5)	4.2 ± 0.4	4.3 (4.0, 4.5)
Ca (mmol/L)	2.4 (2.3, 2.4)	2.3 (2.3, 2.4)	2.4 (2.3, 2.4)
P (mmol/L)	1.2 (1.1, 1.3)	1.2 ± 0.2	1.2 (1.1, 1.3)
TC (mmol/L)	4.8 (4.1, 5.5)	4.8 (4.3, 5.5)	4.8 (4.2, 5.5)
TG (mmol/L)	2.5 (1.7, 3.7)	2.9 (2.0, 4.2)	2.6 (1.8, 3.8)
HDL (mmol/L)	1.0 (0.9, 1.2)	0.9 (0.8, 1.1)	1.0 (0.9, 1.1)
LDL (mmol/L)	2.3 (1.9, 2.8)	2.4 (2.0, 2.8)	2.3 (1.9, 2.8)
Apoa1 (g/L)	1.2 (1.1, 1.3)	1.2 (1.1, 1.3)	1.2 (1.1, 1.3)
Apob (g/L)	1.0 (0.9, 1.2)	1.1 (0.9, 1.3)	1.0 (0.9, 1.2)
UACR (mg/g)	9.9 (6.7, 16.7)	10.0 (6.7, 18.4)	10.0 (6.7, 16.9)
TyG index	9.8 (9.4, 10.3)	10.1 (9.7, 10.4)	9.9 (9.5, 10.3)
TC/HDL	4.9 (3.9, 5.9)	5.1 (4.3, 6.2)	4.9 (4.0, 5.9)
TSH (uIU/ml)	2.0 (1.3, 2.9)	2.0 (1.3, 2.6)	2.0 (1.3, 2.8)
fT3 (pmol/L)	4.3 (3.9, 4.7)	4.2 ± 0.7	4.3 (3.8, 4.7)
fT4 (pmol/L)	16.6 (15.0, 18.3)	16.5 ± 2.2	16.6 (14.9, 18.3)
QRSKI3	7.5 (3.2, 14.4)	23.8 (16.6, 32.6)	10.6 (4.3, 19.5)
QRSIK3 (<10%), n (%)	262.0 (61.2)	12.0 (8.7)	274.0 (48.4)
QRSIK3 (10%-20%), n (%)	128.0 (29.9)	35.0 (25.4)	163.0 (28.8)
QRSIK3 (> 20%), n (%)	38.0 (8.9)	91.0 (65.9)	129.0 (22.8)
FRS	9.7 (4.7, 18.4)	25.3 (15.6, 30.0)	13.3 (5.6, 21.6)
FRS (<10%), n (%)	214.0 (50.0)	15.0 (10.9)	229.0 (40.5)
FRS (10%-20%), n (%)	129.0 (30.1)	41.0 (29.7)	170.0 (30.0)
FRS (> 20%), n (%)	85.0 (19.9)	82.0 (59.4)	167.0 (29.5)

The eGFR is calculated according to the CKD-EPI formula. CVD, cardiovascular disease; BMI, body mass index; SBP, systolic blood pressure; FBG, fasting blood glucose; PBG, postprandial blood glucose; HbA1c, haemoglobin A1c; FCP, fasting c- peptide; WBC, white blood cell; Hb, haemoglobin; NLR, neutrophil to lymphocyte ratio; FIB, fibrinogen; ALT, alanine aminotransferase; AST, aspartate aminotransferase; ALB, serum albumin; BUN, blood urea nitrogen; SCr, serum creatinine; UA, uric acid; CysC, cystatin C; eGFR, estimated glomerular filtration rate; TC, total cholesterol; TG, triglycerides; HDL, high density lipoprotein; LDL, low density lipoprotein; Apoa1, apolipoprotein a1; Apob, apolipoprotein b; UACR, urine albumin-to-creatinine ratio; TyG index, triglyceride-glucose index; TSH, thyroid stimulating hormone; fT3, free triiodothyronine; fT4, free thyroxine; QRISK3, QRESEARCH risk estimator version 3; FRS, framingham risk score.

### Risk factors for CVD

**Table 2** shows risk factors for the prediction of CVD in T2DM patients. In univariate analysis, QRISK3 and FRS were significant risk factors for CVD (*p*<0.001). The results of cox regression analysis showed that QRISK3 (HR=5.972, *p*< 0.001, 95% CI 4.565 to 7.813) and FRS (HR=3.223, *p*< 0.001, 95% CI 2.535 to 4.097) were risk factors for CVD. In addition, age, sex (male), smoking history, hypertension history, SBP, FCP, white blood cell (WBC), fibrinogen (FIB), blood urea nitrogen (BUN), serum creatinine (Scr), uric acid (UA), TyG index and TC/HDL were also risk factors for CVD in patients with T2DM (*p*<0.05). While albumin (ALB) and estimated glomerular filtration rate (eGFR) were protective factors for CVD (*p*<0.05).

**Table 2:** Cox regression analysis of CVD- related risk factors.

Variables	β-coefficient	HR (95% CI)	*P* value
Age	0.087	1.190 (1.074, 1.108)	<0.001^*^
Sex (female)	0.524	1.689 (1.159, 2.463)	0.006^*^
BMI	0.018	1.018 (0.968, 1.070)	0.492
Smoking	0.856	2.374 (1.692, 3.330)	<0.001^*^
Hypertension	1.52	4.571 (3.113, 6.711)	<0.001^*^
SBP	0.018	1.019 (1.009, 1.028)	<0.001^*^
FBG	0.056	1.057 (0.997, 1.121)	0.062
PBG	0.022	1.023 (0.981, 1.067)	0.297
HbA1c	-0.023	0.977 (0.910, 1.049)	0.521
FCP	0.181	1.198 (1.018, 1.410)	0.029^*^
WBC	0.155	1.168 (1.067, 1.278)	0.001^*^
Hb	-0.003	0.997 (0.987, 1.006)	0.484
NLR	-0.001	0.999 (0.988, 1.010)	0.857
FIB	0.209	1.232 (1.037, 1.456)	0.018^*^
ALT	-0.004	0.996 (0.989, 1.002)	0.206
AST	-0.009	0.991 (0.976, 1.005)	0.204
ALB	-0.073	0.930 (0.894, 0.968)	<0.001^*^
Bicarbonate	0.004	1.045 (0.987, 1.107)	0.132
BUN	0.123	1.131 (1.065, 1.201)	<0.001^*^
SC r	0.018	1.019 (1.008, 1.029)	0.001^*^
UA	0.002	1.002 (1.000, 1.004)	0.032*
CysC	0.299	1.349 (0.677, 2.687)	0.394
eGFR	-0.015	0.985 (0.979, 0.991)	<0.001^*^
K	-0.254	0.776 (0.546, 1.102)	0.157
Ca	-0.993	0.370 (0.170, 1.279)	0.116
P	-0.535	0.586 (0.241, 1.423)	0.238
TC	0.044	1.045 (0.913, 1.196)	0.524
TG	0.029	1.030 (0.976, 1.086)	0.284
HDL	-0.71	0.492 (0.240, 1.008)	0.053
LDL	0.062	1.064 (0.839, 1.350)	0.608
Apoa1	0.428	1.534 (0.624, 3.773)	0.352
Apob	0.356	1.428 (0.964, 2.115)	0.076
UACR	0.002	1.002 (0.998, 1.006)	0.392
TyG Index	0.277	1.320 (1.049, 1.659)	0.018^*^
TC/HDL	0.105	1.111 (1.014, 1.216)	0.023^*^
TSH	0.001	1.001 (0.901, 1.112)	0.99
fT3	-0.113	0.875 (0.723, 1.060)	0.172
fT4	-0.04	0.961 (0.903, 1.024)	0.217
QRISK3 (Grade)	1.787	5.972 (4.565, 7.813)	<0.001^*^
FRS (Grade)	1.17	3.223 (2.535, 4.097)	<0.001^*^

CVD, cardiovascular disease; BMI, body mass index; SBP, systolic blood pressure; FBG, fasting blood glucose; PBG, postprandial blood glucose; HbA1c, haemoglobin A1c; FCP, fasting c-peptide; WBC, white blood cell; Hb, haemoglobin; NLR, neutrophil to lymphocyte ratio; FIB, fibrinogen; ALT, alanine aminotransferase; AST, aspartate aminotransferase; ALB, serum albumin; BUN, blood urea nitrogen; SCr, serum creatinine; UA, uric acid; CysC, cystatin C; eGFR, estimated glomerular filtration rate; TC, total cholesterol; TG, triglycerides; HDL, high density lipoprotein; LDL, low density lipoprotein; Apoa1, apolipoprotein a1; Apob, apolipoprotein b; UACR, urine albumin-to-creatinine ratio; TyG index, triglyceride-glucose index; TSH, thyroid stimulating hormone; fT3, free triiodothyronine; fT4, free thyroxine; QRISK3, QRESEARCH risk estimator version 3; FRS, framingham risk score.

### Tool evaluation

**Figure 3:**
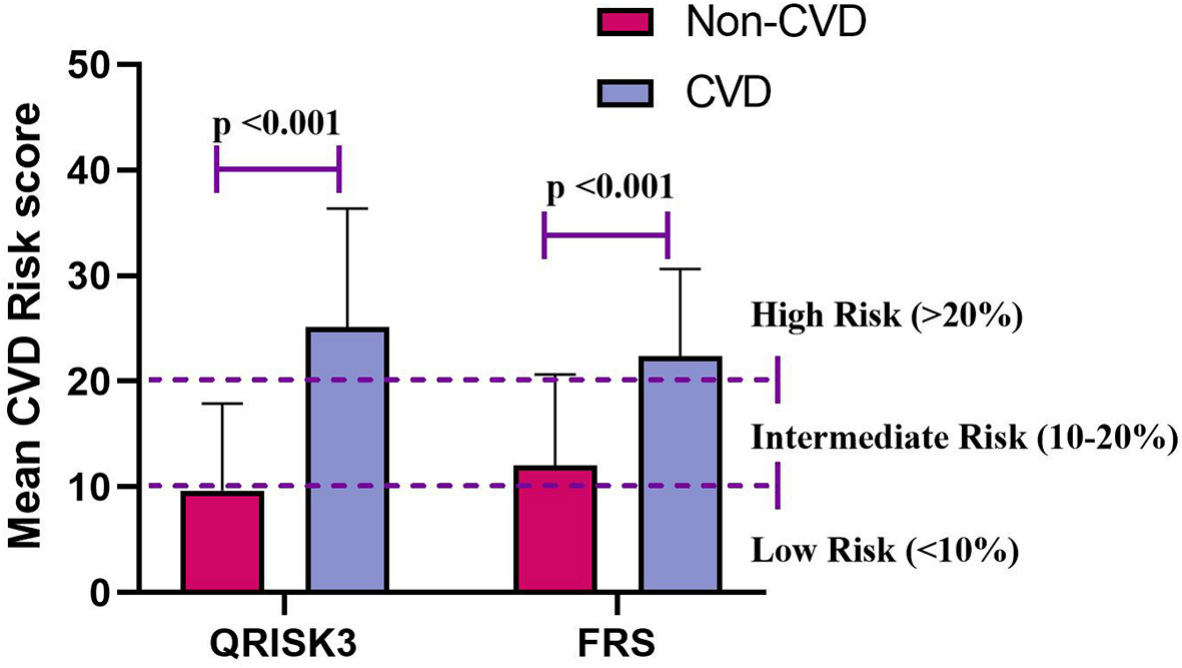
Mean CVD risk score for QRISK3 and FRS. Legend: Stratified according to patients with CVD (n=138) and patients without CVD (n=428). CVD, cardiovascular disease; QRISK3, QRESEARCH risk estimator version 3; FRS, Framingham risk score.

Interestingly, **Table 1** shows that QRSKI3 and FRS scored higher in CVD patients compared with non-CVD patients. The median 10-year CVD risk scores for the QRISK3 and FRS among patients without CVD were 7.5% and 9.7%, respectively (**Figure 3**). However, for CVD patients, the median 10-year CVD risk scores were 23.8% and 25.3%, respectively (**Figure 3**).

**Figure 4:**
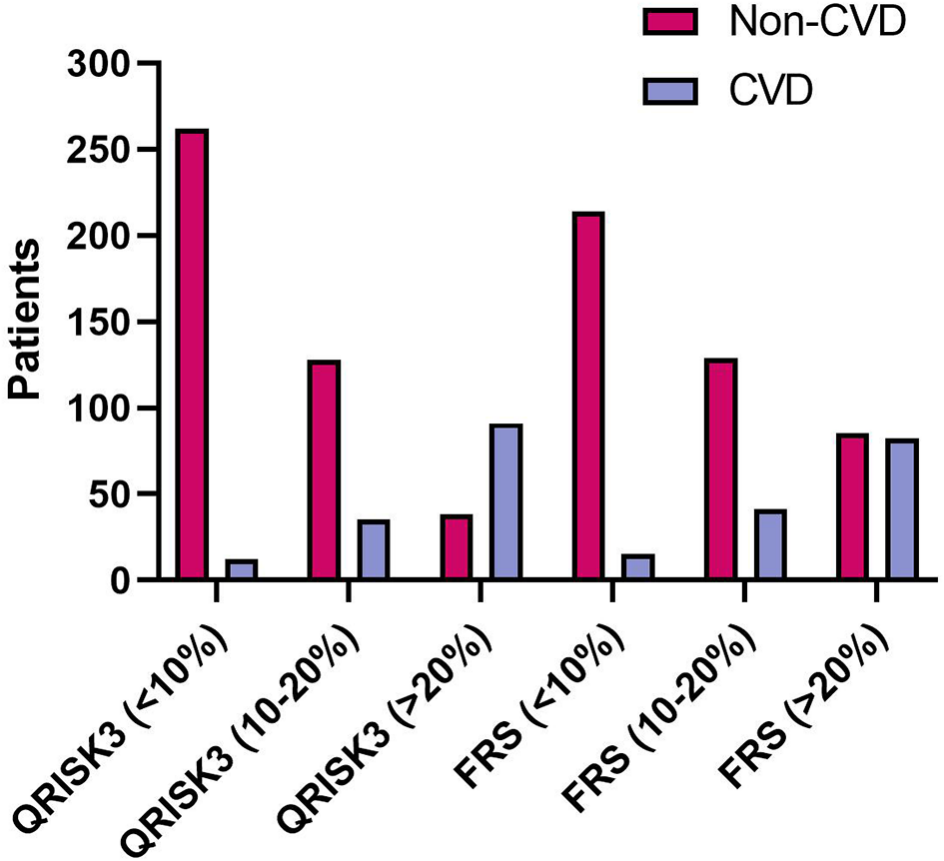
The number of patients considered low (<10%), median (10%–20%) and high (>20%) risk between patients according to QRISK3 and FRS. Legend: QRISK3 identified the majority of non-CVD patients as low or intermediate risk, with 262 (61.2%) patients classified as low risk and 128 (29.9%) patients classified as intermediate risk. Furthermore, QRISK3 identified 91 (65.9%) of CVD patients as high-risk. However, FRS classified some non-CVD patients as high risk. The number of CVD patients classified as high risk was 82 (59.4%), while the number of non-CVD classified as high risk was 85 (61.6%). CVD, cardiovascular disease; QRISK3, QRESEARCH Risk estimator version 3; FRS, Framingham risk score.

When examining risk stratification, QRISK3 classified most non-CVD patients as low and intermediate risk, with 262 (61.2%) patients at low risk, and 128 (29.9%) patients at intermediate risk, respectively (**Figure 4**). Moreover, QRISK3 classified most CVD patients as high-risk, with 91 (65.9%) patients (**Figure 4**). However, FRS classified 85 (61.6%) non-CVD patients as high risk, and the ratio of CVD patients classified as high risk based on FRS was lower than QRISK3 (61.2% vs 59.4%) (**Figure 4**).

**Figure 5:**
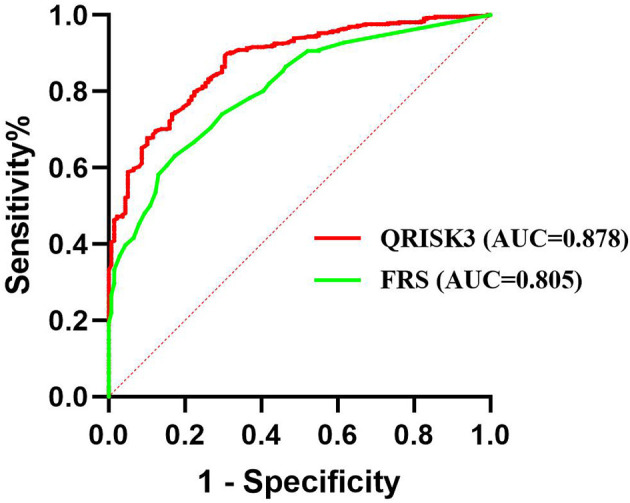
ROC for QRISK3 and FRS. Legend: ROC curves were drawn without distinguishing the risk levels, and the AUC of QRISK3 and FRS were high, which were 0.878 and 0.805, respectively. ROC, Receiver operating characteristics; AUC, area under the ROC curve; QRISK3, QRESEARCH Risk estimator version 3; FRS, Framingham risk score.

To compare the risk prediction between QRISK3 and FRS, AUC analysis was used. The AUC of QRISK3 and FRS were 0.878 and 0.805 (**Figure 5**). Moreover, **Table 3** lists the sensitivity, specificity, PPV, NPV and C-statistics obtained by dichotomizing the risk score using the cut-off of 10% and 20% 10-year CVD for the two tools. The FRS had the best PPV of 93.4% and 86% but the lowest NPV was 36.5 and 49.1%. In addition, with a 10% cut-off dichotomy, FRS had a higher specificity of 89.1%, but a higher sensitivity of 80.1% on 20% cut-off dichotomy. Meanwhile, QRISK3 had a high sensitivity of 91.3% on a 10% cut-off dichotomy, but a higher specificity of 90.7% on a 20% cut-off dichotomy. The C-statistics of QRISK3 in the two cut-off dichotomies were the highest, which were 0.763 and 0.787, respectively (**Table 3**). In addition, we calculated the Kappa values of the total-risk category, low-risk category and high-risk category, which were 0.587, 0.726 and 0.645 respectively (**Table 4**).

**Table 3:** Sensitivity, specificity, PPV, NPV and C-statistics of QRISK3 and FRS.

Tools	Sensitivity (%)	Specificity (%)	PPV (%)	NPV (%)	C-statistics (95% CI)
QRISK3†	91.3	61.2	43.1	95.6	0.763 (0.721, 0.804)
FRS†	50.0	89.1	93.4	36.5	0.696 (0.650, 0.742)
QRISK3††	66.7	90.7	69.7	89.4	0.787 (0.737, 0.837)
FRS††	80.1	59.4	86.0	49.1	0.698 (0.644, 0.751)

†Dichotomised risk scores using a cut-off of 10% 10-year CVD. †† Dichotomised risk scores using a cut-off of 20% 10-year CVD. PPV, positive predictive value; NPV, negative predictive value; C-statistics, concordance statistics; QRISK3, QRESEARCH risk estimator version 3; FRS, framingham risk score.

**Table 4:** Kappa coefficient demonstrating agreement among total risk category, low risk category and high risk category of QRISK3and FRS.

Kappa	QRISK3	FRS	QRISK3†	FRS†	QRISK3††	FRS††
QRISK3	1	0.587				
FRS	0.587	1				
QRISK3†			1	0.726		
FRS†			0.726	1		
QRISK3††					1	0.645
FRS††					0.645	1

†Dichotomised risk scores using a cut-off of 10% 10-year CVD. †† Dichotomised risk scores using a cut- off of 20% 10-year CVD. QRISK3, QRESEARCH risk estimator version 3; FRS, framingham risk score.

### Sensitivity analyses

**Figure 6:**
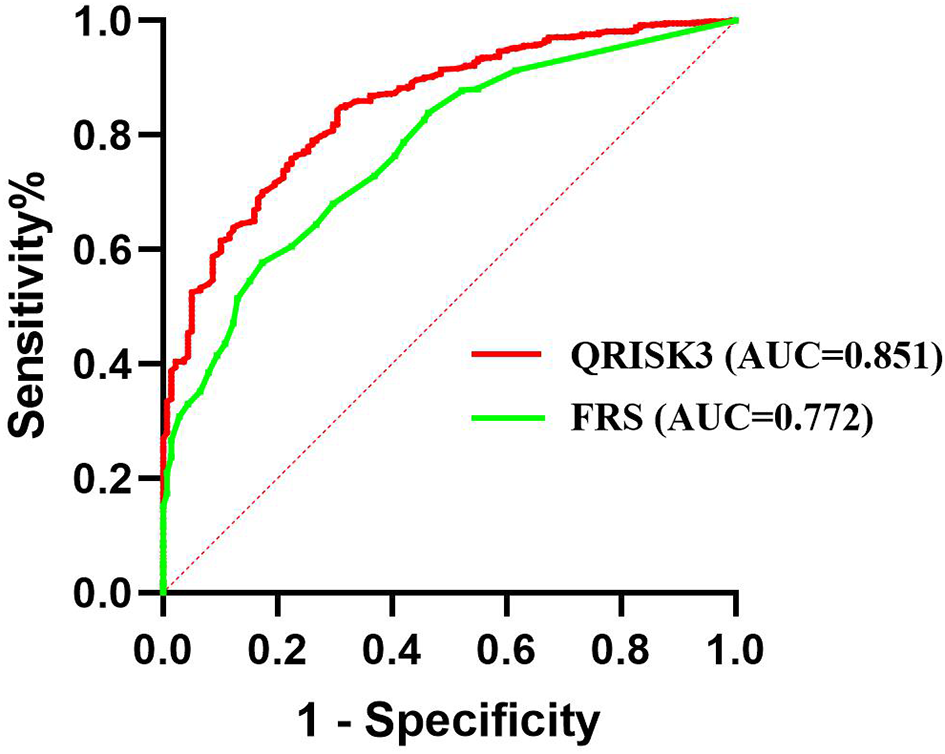
ROC for QRISK3 and FRS (patients with loss to follow-up were classified as non-CVD groups). Legend: All the patients who lost to follow-up were classified as non-CVD group. ROC, Receiver operating characteristics; AUC, area under the ROC curve; QRISK3, QRESEARCH Risk estimator version 3; FRS, Framingham risk score; CVD, cardiovascular disease.

**Figure 7:**
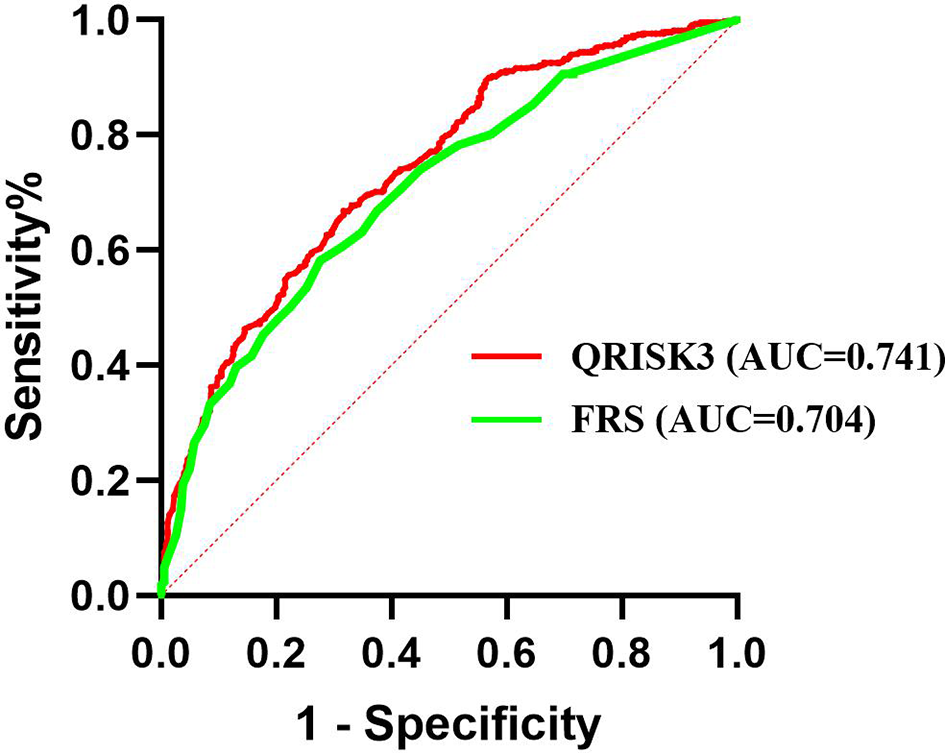
ROC for QRISK3 and FRS (patients with loss to follow-up were classified as CVD groups). Legend: All the patients who lost to follow-up were classified as CVD group. ROC, Receiver operating characteristics; AUC, area under the ROC curve; QRISK3, QRESEARCH Risk estimator version 3; FRS, Framingham risk score; CVD, cardiovascular disease.

We conducted sensitivity analyses to assess the impact of loss to follow up on the results. The results of the number of patients who lost to follow-up were re-analyzed and compared by non-CVD and CVD, respectively. In both different endings, the AUC of QRISK3 and FRS were within the same range and performed well (**Figures 6**, **7**). When patients with loss to follow-up were defined as non-CVD and CVD groups, the AUC of QRISK3 was still higher than FRS. In addition, there was no significant difference in PPV, NPV and C-statistics analysis based on the analysis of the two outcomes (**Tables 5**, **6**). Hence, the loss to the follow-up population did not have a significant impact on the performance of QRISK3 and FRS.

**Table 5:** Sensitivity, specificity, PPV, NPV and C-statistics of QRISK3 and FRS (patients with loss to follow-up were classified as the non-CVD group).

Tools	Sensitivity (%)	Specificity (%)	PPV (%)	NPV (%)	C-statistics (95% CI)
QRISK3†	54.7	91.3	96.7	30.5	0.730 (0.690, 0.770)
FRS†	43.5	89.1	94.8	25.6	0.663 (0.619, 0.708)
QRISK3††	86.1	65.9	92.1	50.8	0.760 (0.711, 0.810)
FRS††	76.3	59.4	89.6	35.3	0.679 (0.627, 0.731)

†Dichotomised risk scores using a cut-off of 10% 10-year CVD. †† Dichotomised risk scores using a cut- off of 20% 10-year CVD. PPV, positive predictive value; NPV, negative predictive value; C-statistics, concordance statistics; QRISK3, QRESEARCH risk estimator version 3; FRS, framingham risk score.

**Table 6:** Sensitivity, specificity, PPV, NPV and C-statistics of QRISK3 and FRS (patients with loss to follow-up were classified as the CVD group).

Tools	Sensitivity (%)	Specificity (%)	PPV (%)	NPV (%)	C-statistics (95% CI)
QRISK3†	59.4	93.6	96.7	42.6	0.665 (0.627, 0.704)
FRS†	50	77.6	73.5	55.5	0.638 (0.599, 0.677)
QRISK3††	93.5	75.0	92.1	78.8	0.661(0.621, 0.700)
FRS††	80.1	42.7	63.5	63.4	0.614 (0.574, 0.655)

†Dichotomised risk scores using a cut- off of 10% 10-year CVD. †† Dichotomised risk scores using a cut-off of 20% 10-year CVD. PPV, positive predictive value; NPV, negative predictive value; C-statistics, concordance statistics; QRISK3, QRESEARCH risk estimator version 3; FRS, framingham risk score.

## Discussion

Studies demonstrated that abnormal glucose metabolism can hasten the formation of atherosclerotic plaque, promote plaque rupture and thrombosis, impair normal endothelial function, and result in CVD events (19). As a result, the risk of CVD grows fast in T2DM patients, and the accompanying CVD classification and prediction tools are essential in assisting patients in risk stratification and guiding preventive therapy (20–22). Using up to 21 clinical risk indicators, QRISK3 is a novel algorithm model based on QRISK2 that forecasts 10-year CVD risk. FRS is a tool that has been widely used in the clinical prediction of 10-year CVD risk.

To our knowledge, this is the first preliminary study to assess QRISK3 in predicting CVD outcomes in the T2DM cohort in China. Our study was a single-center analysis of prospectively collected data from 566 newly-diagnosed T2DM patients, 138 of whom had a CVD event. The QRISK3 algorithm calculates a person’s risk of developing a heart attack or stroke over the next 10 years. It displays the typical risk of individuals who share the same risk factors as those entered for that individual (23). In addition, QRISK3 was widely used to estimate CVD risk in SLE and RA (23, 24). However, only a few studies reported the performances of QRSKI3 in calculating CVD risk related to T2DM (25, 26). In this study, we examined whether QRISK3, as CVD risk tools, can accurately predict CVD in patients with T2DM, and compared its predictive performance with FRS. The results of the univariate analysis were similar to the common CVD risk factors reported in previous literature (age, male, smoking history, hypertension history, etc.) (27). Additionally, there were several risk variables for CVD that were exclusive to DM individuals, such as FCP and TyG index. Compared with non-CVD patients, CVD patients had higher FCP and TyG index. The results were similar to previous literature (19, 28–30). FCP is a molecular peptide separated from proinsulin, which reflects the function of insulin secretion by islet cells (31). TyG index, a viable alternative indicator of insulin resistance, combines triglyceride and fasting blood glucose levels and is significantly related to insulin resistance (32). Studies revealed that a higher TyG index is linked to a higher risk of CVD, and it was shown that insulin resistance is associated with the pathophysiology of the disease (33). There was no difference in hemoglobin A1c (HbA1c) and blood glucose between CVD and non-CVD groups. It might be related to the fact that the subjects were all newly-diagnosed as T2DM patients, so the blood glucose levels in the past three months would not be very different.

QRISK3 and FRS were reliable tools in predicting CVD in T2DM patients, according to the results of the AUC. QRISK3 had superior overall prediction performance than FRS. First, the C-statistic of QRISK3 was the highest no matter which cut-off dichotomy method was used. Second, they performed differently, though, depending on the level of risk stratification at which the sensitivity and specificity were determined. FRS had a high specificity on a 10% cut-off dichotomy and a high sensitivity on a 20% cut-off dichotomy. Furthermore, the NPV of FRS performed poorly, but PPV performed excellently because it identified the most proportion of patients as high-risk and maximized the number of false positives for high CVD risk. However, the NPV of QRISK3 performed well at both the 10% and 20% cut-off dichotomy. QRISK3 had higher specificity based on a 20% cut-off dichotomy and higher sensitivity based on a 10% cut-off dichotomy.

We also demonstrated a discrepancy between the two tools in predicting CVD outcomes. In the low-risk and high-risk categories, QRISK3 and FRS showed higher consistency. But the consistency calculated by the total risk category only showed a ‘moderate’ level of agreement. Thus, the two tools for identifying high-risk and low-risk individuals had similar consistency. But the predictive performance of intermediate-risk people was relatively poor. Early identification of intermediate-risk people may have direct consequences for preventative treatment and cause disagreement in the risk management of physicians treating patients with T2DM. Therefore, further large sample-size studies are needed to verify the predictive power of QRISK3 in intermediate-risk population.

Limited studies explored the roles of both tools on CVD outcomes in T2DM. We conducted a comparative study of the predictive performance of QRISK3 and FRS for CVD events in a large number of newly-diagnosed T2DM patients over a follow-up period of almost 10 years in China. This study also has several limitations. First, since this is a prospectively gathered single-center retrospective cohort study, it is not as scientifically valid as a clinical trial or prospective study, and it may suffer from recollection bias and loss of follow-up. Fortunately, the results of sensitivity analysis showed that the lost population did not have a significant impact on the predictive performance of the two tools. Subjects were recruited in the third affiliated hospital of soochow university, hence it was unable to determine how representative this sample was in the overall T2DM community. Second, data on ‘erectile dysfunction’ were missing from the QRISK3 score. The QRISK3 score has a low weight for ‘erectile dysfunction’ when calculating the 10-year CVD risk, which is relatively insignificant. Finally, other limitations may emerge from potential confounders, such as the use of numerous medicines in individuals with more clinical comorbidities, side effects, or interactions that might alter our results.

## Conclusion

In conclusion, this study supports the claim that QRISK3 is a good predictor of CVD events in T2DM patients during follow-up. QRISK3 has a better predictive ability than FRS in these subjects. In the future, we need to integrate large-scale T2DM cohort studies to further verify the relevant tools for predicting 10-year CVD risk, including QRISK3 and FRS. More research and optimization are needed to develop new or improved CVD risk prediction tools.

## Data availability statement

The original contributions presented in the study are included in the article/**Supplementary Material**. Further inquiries can be directed to the corresponding authors.

## Ethics statement

This research study was approved by the Ethics Committee of the Third Affiliated Hospital of Soochow University (2013#27). The patients/participants provided their written informed consent to participate in this study.

## Author contributions

Conceptualization, HZ and MY. Writing—original draft preparation, XM. Supervision, AW and HH. Funding acquisition, HZ. All authors have read and agreed to the published version of the manuscript. All authors were involved in the study conception and design, acquisition of data, and analysis and interpretation of data.

## Funding

This work was supported by grants from the National Natural Science Foundation of China (82000684) and Changzhou Sci & Tech Program (CJ20200025).

## Conflict of interest

The authors declare that the research was conducted in the absence of any commercial or financial relationships that could be construed as a potential conflict of interest.

## Publisher’s note

All claims expressed in this article are solely those of the authors and do not necessarily represent those of their affiliated organizations, or those of the publisher, the editors and the reviewers. Any product that may be evaluated in this article, or claim that may be made by its manufacturer, is not guaranteed or endorsed by the publisher.

## Abbreviations

ALB, albumin; BUN, blood urea nitrogen; CVD, cardiovascular disease; CVA, cerebrovascular accident; CHD, coronary heart disease; C-statistics, concordance statistics; DM, diabetes mellitus; eGFR, estimated glomerular filtration rate; FRS, Framingham Risk Score; FCP, fast C-peptide; FIB, fibrinogen; HDL, high-density lipoprotein; HR, hazard ratio; IBD, inflammatory bowel disease; MI, myocardial infarction; NPV, negative predictive value; QRISK3, QRESEARCH risk estimator version 3; PAD, peripheral artery disease; RA, rheumatoid arthritis; PPV, positive predictive value; SLE, systemic lupus erythematosus; SBP, systolic blood pressure; Scr, serum creatinine; T2DM, type 2 diabetes mellitus; TIA, transient ischaemic attack; TyG index, triglyceride-glucose index; WBC, white blood cell.

## References

[1] Cole JB Florez JC. Genetics of diabetes mellitus and diabetes complications. Nat Rev Nephrol (2020) 16(7):377–90. doi: 10.1038/s41581-020-0278-5 PMC9639302 32398868

[2] Li Y Teng D Shi X Qin G Qin Y Quan H. Prevalence of diabetes recorded in mainland China using 2018 diagnostic criteria from the American diabetes association: National cross sectional study. BMJ (Clinical Res ed) (2020) 369:m997. doi: 10.1136/bmj.m997 PMC7186854 32345662

[3] Glovaci D Fan W Wong ND. Epidemiology of diabetes mellitus and cardiovascular disease. Curr Cardiol Rep (2019) 21(4):21. doi: 10.1007/s11886-019-1107-y 30828746

[4] Dal Canto E Ceriello A Rydén L Ferrini M Hansen TB Schnell O. Diabetes as a cardiovascular risk factor: An overview of global trends of macro and micro vascular complications. Eur J Prev Cardiol (2019) 26(2_suppl):25–32. doi: 10.1177/2047487319878371 31722562

[5] Rao Kondapally Seshasai S Kaptoge S Thompson A Di Angelantonio E Gao P Sarwar N. Diabetes mellitus, fasting glucose, and risk of cause-specific death. New Engl J Med (2011) 364(9):829–41. doi: 10.1056/NEJMoa1008862 PMC4109980 21366474

[6] Sarwar N Gao P Seshasai SRK Gobin R Kaptoge S Di Angelantonio E. Diabetes mellitus, fasting blood glucose concentration, and risk of vascular disease: a collaborative meta-analysis of 102 prospective studies. Lancet (2010) 375(9733):2215–22. doi: 10.1016/S0140-6736(10)60484-9 PMC2904878 20609967

[7] Whyte MB Joy M Hinton W McGovern A Hoang U van Vlymen J. Early and ongoing stable glycaemic control is associated with a reduction in major adverse cardiovascular events in people with type 2 diabetes: A primary care cohort study. Diabetes Obes Metab (2022) 24(7):1310–18. doi: 10.1111/dom.14705 PMC9320871 35373891

[8] Haas AV McDonnell ME. Pathogenesis of cardiovascular disease in diabetes. Endocrinol Metab Clin North Am (2018) 47(1):51–63. doi: 10.1016/j.ecl.2017.10.010 29407056

[9] Hippisley-Cox J Coupland C Brindle P. Development and validation of QRISK3 risk prediction algorithms to estimate future risk of cardiovascular disease: Prospective cohort study. BMJ (Clinical Res ed) (2017) 357:j2099. doi: 10.1136/bmj.j2099 PMC5441081 28536104

[10] Livingstone SJ Guthrie B Donnan PT Thompson A Morales DR. Predictive performance of a competing risk cardiovascular prediction tool CRISK compared to QRISK3 in older people and those with comorbidity: Population cohort study. BMC Med (2022) 20(1):152. doi: 10.1186/s12916-022-02349-6 35505353 PMC9066924

[11] Hippisley-Cox J Coupland C Brindle P. The performance of seven QPrediction risk scores in an independent external sample of patients from general practice: A validation study. BMJ Open (2014) 4(8):e005809. doi: 10.1136/bmjopen-2014-005809 PMC4156807 25168040

[12] Sivakumaran J Harvey P Omar A Tayer-Shifman O Urowitz MB Gladman DD. Assessment of cardiovascular risk tools as predictors of cardiovascular disease events in systemic lupus erythematosus. Lupus Sci Med (2021) 8(1):e000448. doi: 10.1136/lupus-2020-000448 34045359 PMC8162102

[13] Quevedo-Abeledo JC Caceres L Palazuelos C Llorca J González-Gay MÁ Ferraz-Amaro I. QRISK3 relation to carotid plaque is higer than that of score in patients with systemic lupus erythematosus. Rheumatol (Oxford) (2022) 61(4):1408–16. doi: 10.1093/rheumatology/keab531 34240117

[14] D’Agostino RB Vasan RS Pencina MJ Wolf PA Cobain M Massaro JM. General cardiovascular risk profile for use in primary care: the framingham heart study. Circulation (2008) 117(6):743–53. doi: 10.1161/CIRCULATIONAHA.107.699579 18212285

[15] Ko DT Sivaswamy A Sud M Kotrri G Azizi P Koh M. Calibration and discrimination of the framingham risk score and the pooled cohort equations. CMAJ (2020) 192(17):E442–E49. doi: 10.1503/cmaj.190848 PMC7207198 32392491

[16] Petruzzo M Reia A Maniscalco GT Luiso F Lanzillo R Russo CV. The framingham cardiovascular risk score and 5-year progression of multiple sclerosis. Eur J Neurol (2021) 28(3):893–900. doi: 10.1111/ene.14608 33091222

[17] Association AD. 11. microvascular complications and foot care: Standards of medical care in diabetes-2019. Diabetes Care (2019) 42(Suppl 1):S124–S38. doi: 10.2337/dc19-S011 30559237

[18] Akobeng AK. Understanding diagnostic tests 3: Receiver operating characteristic curves. Acta Paediatr (2007) 96(5):644–47. doi: 10.1111/j.1651-2227.2006.00178.x 17376185

[19] Su W-Y Chen S-C Huang Y-T Huang J-C Wu P-Y Hsu W-H. Comparison of the effects of fasting glucose, hemoglobin a, and triglyceride-glucose index on cardiovascular events in type 2 diabetes mellitus. Nutrients (2019) 11(11):2838. doi: 10.3390/nu11112838 31752391 PMC6893677

[20] Damen JAAG Hooft L Schuit E Debray TPA Collins GS Tzoulaki I. Prediction models for cardiovascular disease risk in the general population: systematic review. BMJ (Clinical Res ed) (2016) 353:i2416. doi: 10.1136/bmj.i2416 PMC4868251 27184143

[21] Damen JA Pajouheshnia R Heus P Moons KGM Reitsma JB Scholten RJPM. Performance of the framingham risk models and pooled cohort equations for predicting 10-year risk of cardiovascular disease: a systematic review and meta-analysis. BMC Med (2019) 17(1):109. doi: 10.1186/s12916-019-1340-7 31189462 PMC6563379

[22] Zhiting G Jiaying T Haiying H Yuping Z Qunfei Y Jingfen J. Cardiovascular disease risk prediction models in the Chinese population- a systematic review and meta-analysis. BMC Public Health (2022) 22(1):1608. doi: 10.1186/s12889-022-13995-z 35999550 PMC9400257

[23] Corrales A Vegas-Revenga N Atienza-Mateo B Corrales-Selaya C Prieto-Peña D Rueda-Gotor J. Combined use of QRISK3 and SCORE as predictors of carotid plaques in patients with rheumatoid arthritis. Rheumatol (Oxford) (2021) 60(6):2801–07. doi: 10.1093/rheumatology/keaa718 33249513

[24] Drosos GC Konstantonis G Sfikakis PP Tektonidou MG. Underperformance of clinical risk scores in identifying vascular ultrasound-based high cardiovascular risk in systemic lupus erythematosus. Eur J Prev Cardiol (2020) 2047487320906650. doi: 10.1177/2047487320906650 32122200

[25] Khanna NN Jamthikar AD Gupta D Nicolaides A Araki T Saba L. Performance evaluation of 10-year ultrasound image-based stroke/cardiovascular (CV) risk calculator by comparing against ten conventional CV risk calculators: A diabetic study. Comput Biol Med (2019) 105:125–43. doi: 10.1016/j.compbiomed.2019.01.002 30641308

[26] Dziopa K Asselbergs FW Gratton J Chaturvedi N Schmidt AF. Cardiovascular risk prediction in type 2 diabetes: a comparison of 22 risk scores in primary care settings. Diabetologia (2022) 65(4):644–56. doi: 10.1007/s00125-021-05640-y PMC8894164 35032176

[27] Tai S Fu L Zhang N Yang R Zhou Y Xing Z. Association of the cumulative triglyceride-glucose index with major adverse cardiovascular events in patients with type 2 diabetes. Cardiovasc Diabetol (2022) 21(1):161. doi: 10.1186/s12933-022-01599-1 35999546 PMC9400318

[28] Wang L Cong H-L Zhang J-X Hu Y-C Wei A Zhang Y-Y. Triglyceride-glucose index predicts adverse cardiovascular events in patients with diabetes and acute coronary syndrome. Cardiovasc Diabetol (2020) 19(1):80. doi: 10.1186/s12933-020-01054-z 32534586 PMC7293784

[29] Park K Ahn CW Lee SB Kang S Nam JS Lee BK. Elevated TyG index predicts progression of coronary artery calcification. Diabetes Care (2019) 42(8):1569–73. doi: 10.2337/dc18-1920 31182490

[30] Li H Zuo Y Qian F Chen S Tian X Wang P. Triglyceride-glucose index variability and incident cardiovascular disease: A prospective cohort study. Cardiovasc Diabetol (2022) 21(1):105. doi: 10.1186/s12933-022-01541-5 35689232 PMC9188105

[31] Huang Y Wang Y Liu C Zhou Y Wang X Cheng B. C-peptide, glycaemic control, and diabetic complications in type 2 diabetes mellitus: A real-world study. Diabetes Metab Res Rev (2022) 38(4):e3514. doi: 10.1002/dmrr.3514 34841643

[32] Ding X Wang X Wu J Zhang M Cui M. Triglyceride-glucose index and the incidence of atherosclerotic cardiovascular diseases: a meta-analysis of cohort studies. Cardiovasc Diabetol (2021) 20(1):76. doi: 10.1186/s12933-021-01268-9 33812373 PMC8019501

[33] Alizargar J Bai C-H Hsieh N-C Wu S-FV. Use of the triglyceride-glucose index (TyG) in cardiovascular disease patients. Cardiovasc Diabetol (2020) 19(1):8. doi: 10.1186/s12933-019-0982-2 31941513 PMC6963998

